# Supplementary figures and images for: Prmt5 promotes vascular morphogenesis independently of its methyltransferase activity
Source: PLoS Genet. 2021 Jun 21;17(6):e1009641. doi: 10.1371/journal.pgen.1009641 (PMC8248709; doi:10.1371/journal.pgen.1009641)

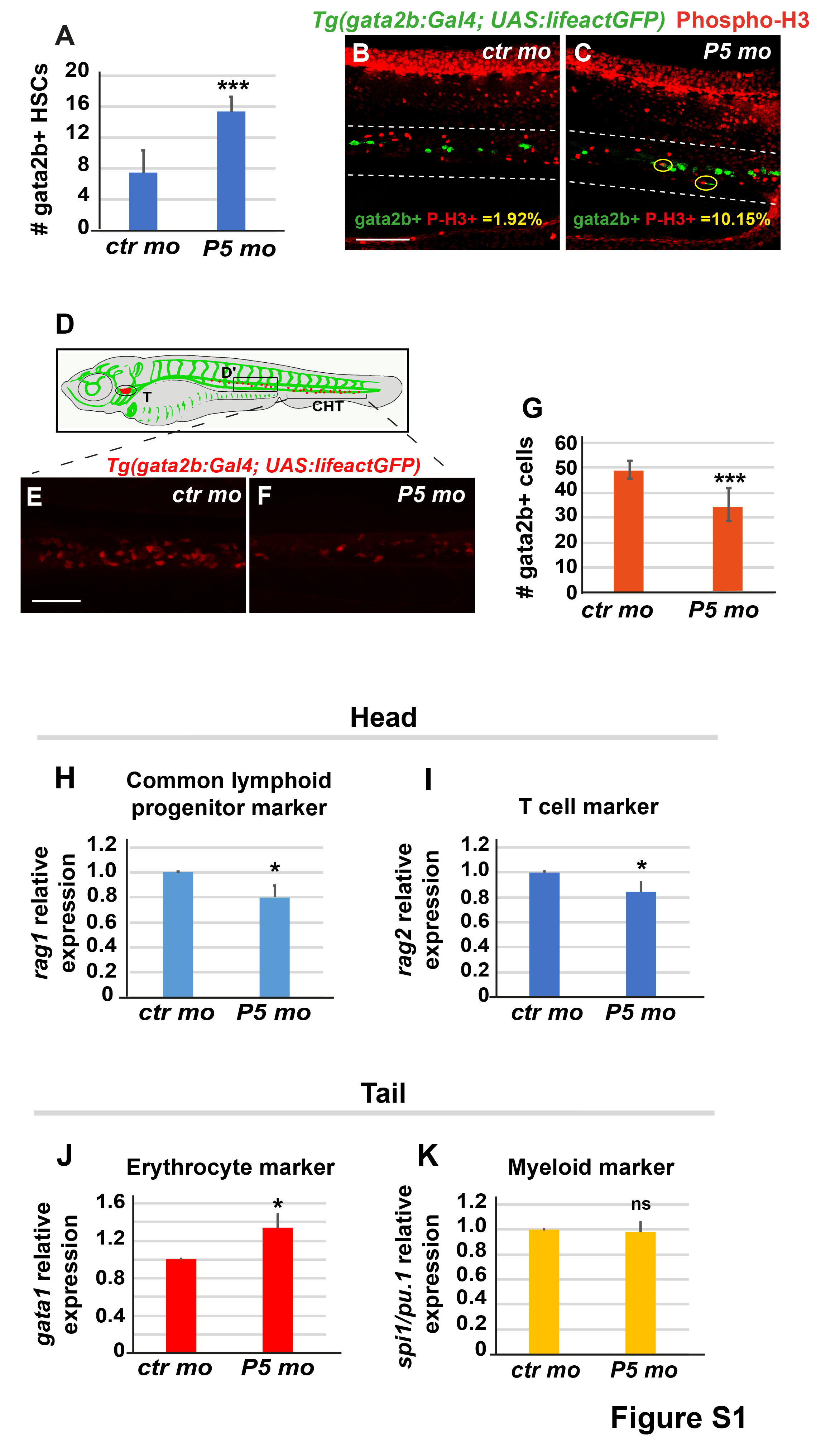

Supplement: S1 Fig — (A) Average number of HSCs enumerated per confocal stack in control and in prmt5 morphant embryos at 40 hpf. Data are from 2 independent experiments with at least 3 individuals per experiment and a t-test was performed. *** P<0,001. (B, C) Confocal projections of immunostaining with anti-phosphoH3 antibody (in red) of Tg(gata2b:Gal4; UAS:lifeactGFP) transgenic embryos either injected with control morpholino (B) or prmt5 morpholino (C) at 40 hpf. Scale bar 100 μm. (D) Schematic representation of vascular (green) and hematopoietic (red) systems in a zebrafish larva. Bracket indicate the Caudal Hematopoietic Tissue (CHT). (E, F) Confocal projections of CHT from transgenic Tg(gata2b:Gal4; UAS:lifeactGFP) embryos at 3 days in the same conditions as in B-C. Scale bar 100 μm. (G) Average number of HSPCs enumerated per confocal stack in control and prmt5 morphant embryos at 3 days from 2 independent experiments with at least 4 individuals per analysis. T-test was performed. *** P<0.001. (H-K) Relative mRNA expressions determined by RT-qPCR in either the head (H, I) or the trunk (J, K) of 3-day old control and prmt5 morphant embryos, from 4 independent experiments with at least 10 animals per condition. T-test was performed. * P<0,05. (TIF) [file pgen.1009641.s001.tif]

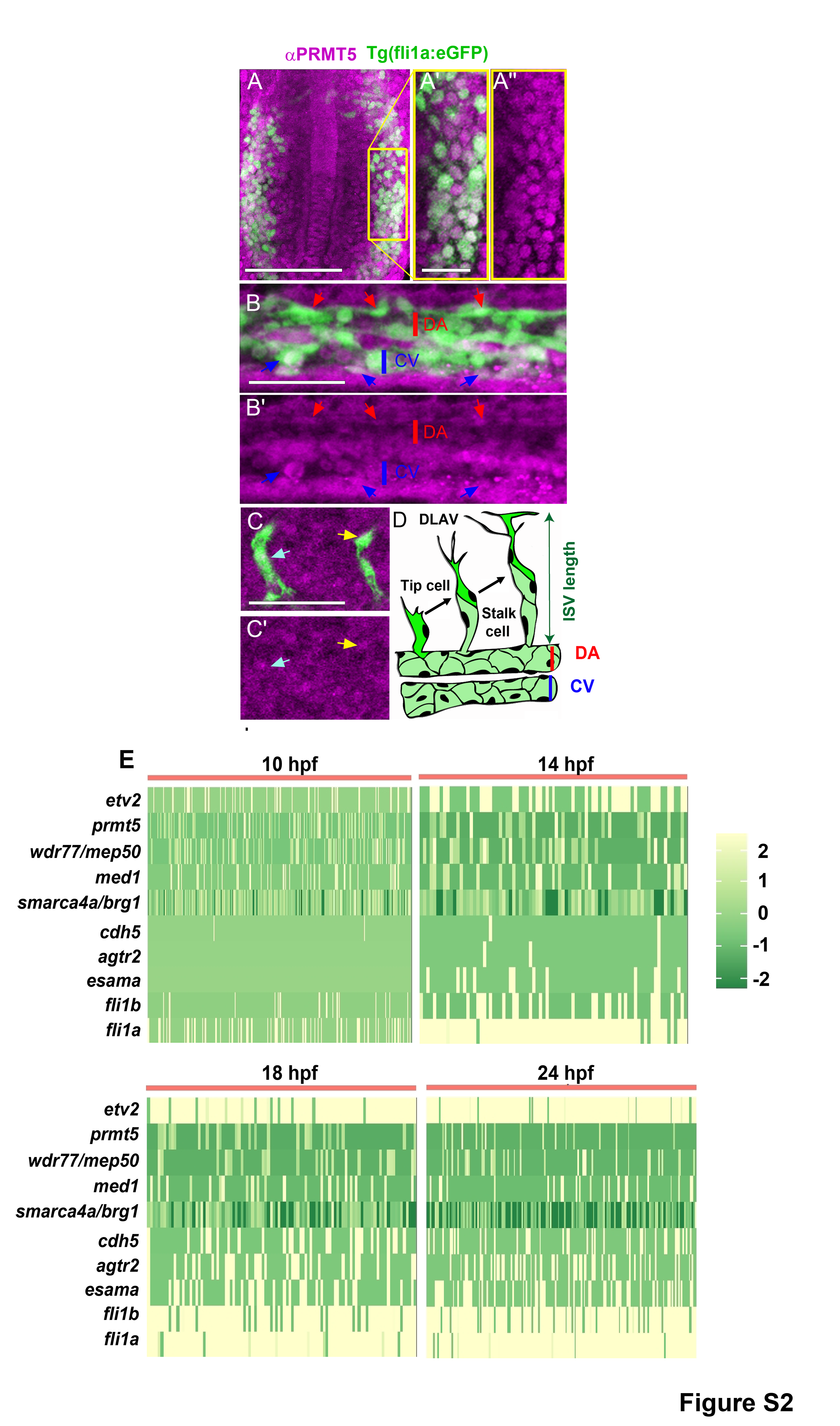

Supplement: S2 Fig — (A-C’) Confocal projections of transgenic Tg(fli1a:GFP)y1 embryos with endothelial cells (in green) after immunostaining against Prmt5 (in magenta). (A-A”) Dorsal view of the lateral plate mesoderm at 14 somite- stage. Yellow rectangle delimits the close up of Prmt5+ endothelial cells (A’-A”). Prmt5+ cells appear in magenta (A-A”) and endothelial cells in green (A-A’). Anterior is on top. Scale bars 100 μm (A) and 25 μm (A’). (B, B’) Confocal projections focusing on endothelial cells (in green) from the dorsal aorta (DA) and the cardinal vein (CV) at 24 hpf. Red and blue arrows point to Prmt5+ cells (in magenta) from the DA and the CV, respectively. Red and blue lines represent DA and CV diameters, respectively. Scale bar 50 μm. (C, C’) Confocal projections focusing on sprouting ISVs (in green) at 24 hpf. Light blue and yellow arrows point to tip and stalk cell, respectively. (D) Schematic representation of the trunk vasculature with ISVs sprouting from the DA. The tip cell leads the cell migration and the stalk cell maintains the connection with the DA. (E) Expression heatmaps for etv2, prmt5, MEP50/Wdr77, Smarca4a/Brg1, MED1 and identified Prmt5 target genes, in endothelial cells at 10 hpf, 14 hpf, 18 hpf and 24 hpf. Data were derived from single cell RNA-sequencing from Wagner et al. [33]. The expression level is colored-coded from absence of expression (green) to highest level of expression (white). (TIF) [file pgen.1009641.s002.tif]

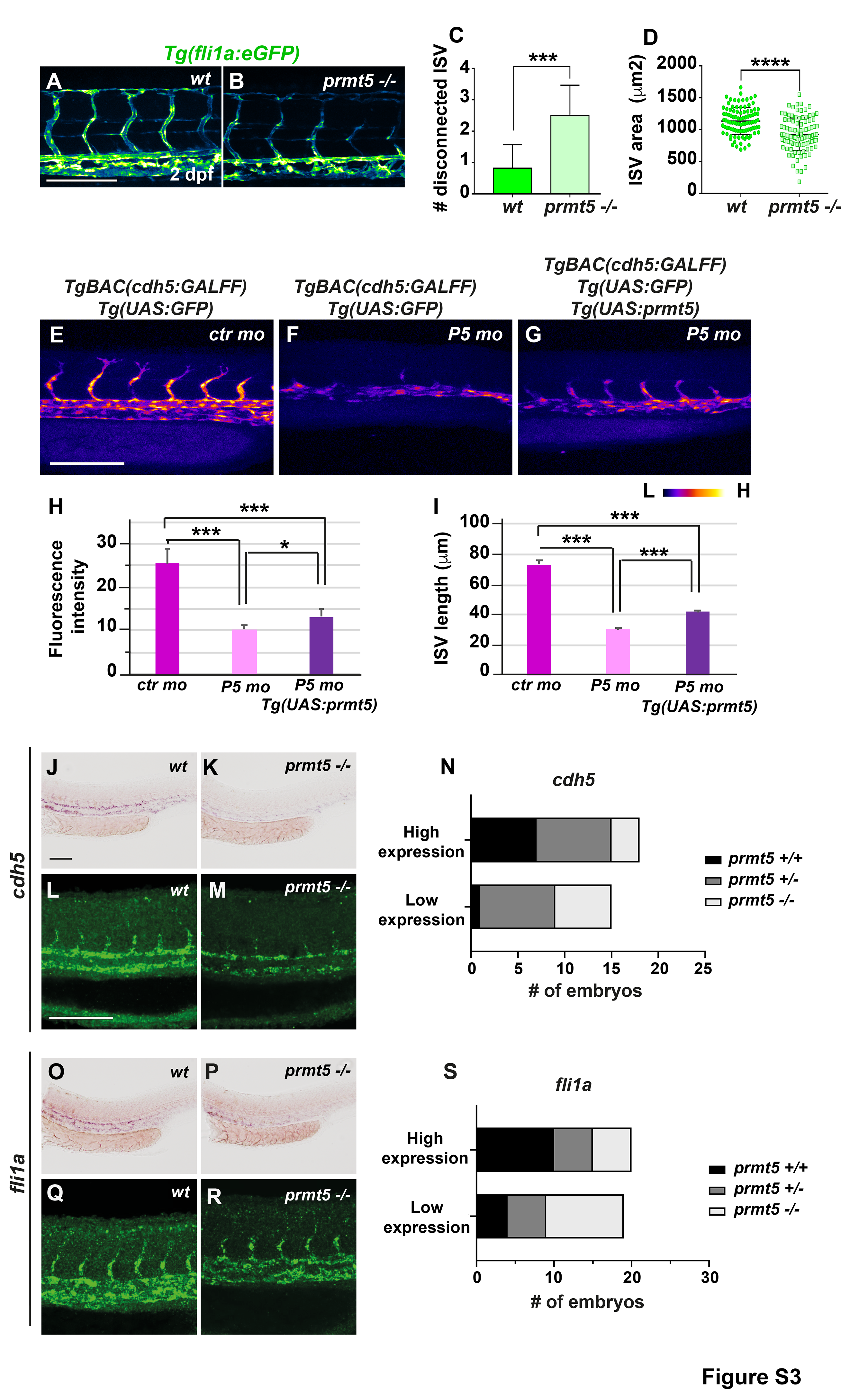

Supplement: S3 Fig — (A, B) Confocal projections of transgenic Tg(fli1a:GFP)y1 wild-type (A) and prmt5 mutant (B) embryos at 48 hpf. Scale bar 100 μm. (C, D) Average number of disconnected ISV per confocal projection (C) and average ISV area in μm2 (D) in control and prmt5 mutant embryos, from 3 independent experiments with at least 4 animals per condition. Mann-Whitney tests were performed. **** P<0.0001; *** P<0.001. (E-G) Confocal projections of transgenic TgBAC(cdh5:GAL4FF);Tg(UAS:GFP) and TgBAC(cdh5:GAL4FF);Tg(UAS:GFP); Tg(UAS:prmt5) embryos at 28 hpf. Control morphant is on the top left panel (E), prmt5 morphant embryos not expressing (F) or mis-expressing prmt5WT in blood vessel (G). The fluorescent intensity is colored-coded, from Low intensity (L) in black to High intensity (H) in white. Scale bar 100 μm. (H, I) Average GFP fluorescence intensity per confocal projection (H) and average ISV length in μm (I), for control, prmt5 morphant embryos not expressing or mis-expressing prmt5WT in blood vessels, from 2 independent experiments with at least 3 animals per condition. One-way ANOVA and Kruskal-Wallis test were performed. * P<0.05, *** P<0.001. (J-R) DIC images and confocal projections of wild-type (J,L,O,Q) and prmt5 mutant embryos (K,M,P,R) after in situ hybridization or fluorescent in situ hybridization against cdh5 (J-M) and fli1a (O-R). Scale bar 100 μm. (N, S) Percentage of embryos (y axis) presenting a high or a low level of expression of cdh5 (N) or fli1a (S), according to their genotype (x axis), from 3 independent experiments with at least 4 animals per condition. (TIF) [file pgen.1009641.s003.tif]

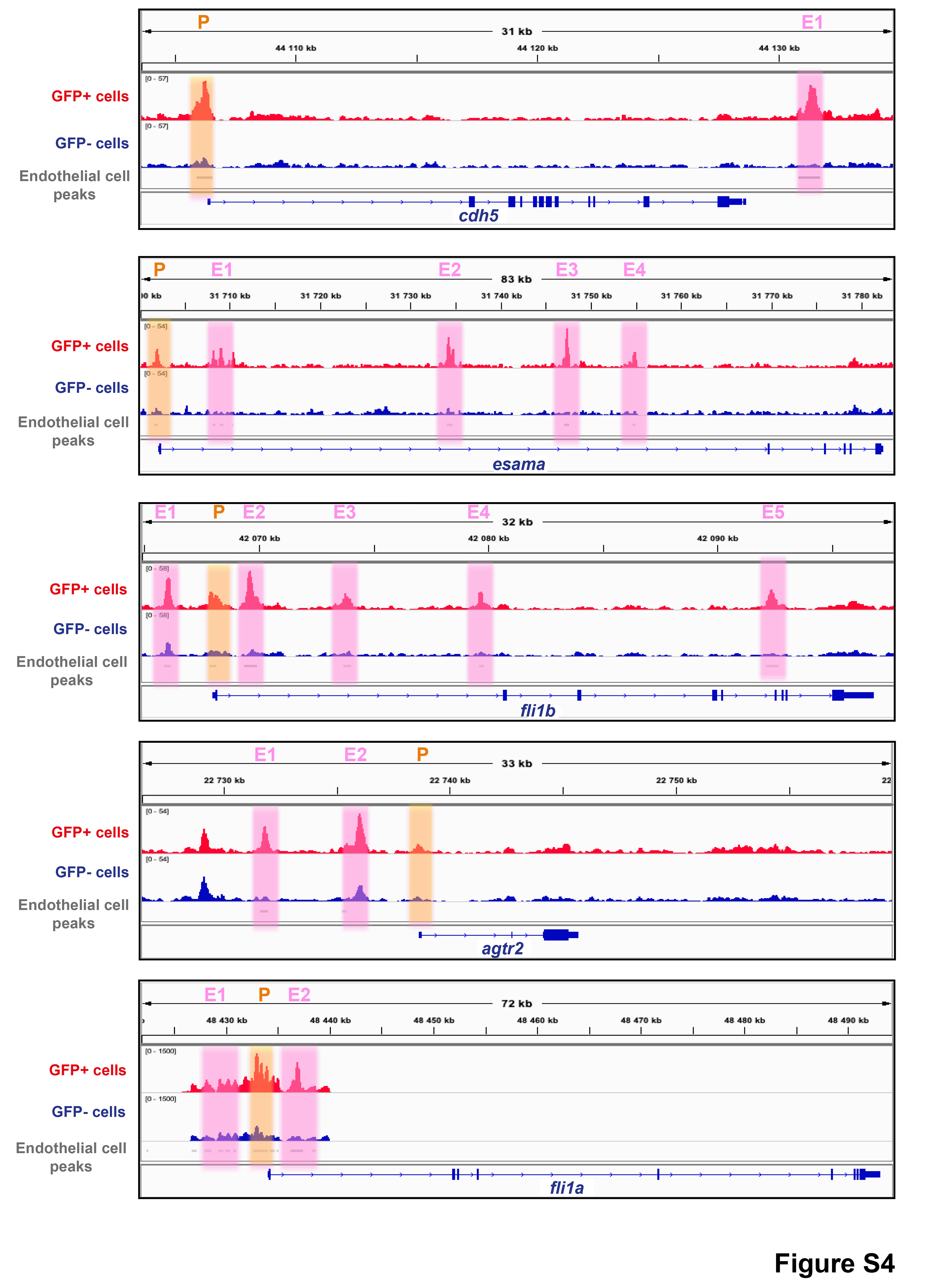

Supplement: S4 Fig — ATAC-seq peaks as determined by Quillien et al. [38] flanking potential Etv2 target genes as indicated (cdh5, esama, fli1b, atgr2, fli1a). GFP+ cell track corresponds to endothelial cell ATAQ-seq profile and GFP-minus track represents non-endothelial cell profile. Endothelial cell peak track shows specific peaks for endothelial cells. Promoter regions (P) and putative enhancers (E) are highlighted in light orange and light pink, respectively. (TIF) [file pgen.1009641.s004.tif]
