# Supplementary material for: Prmt5 promotes vascular morphogenesis independently of its methyltransferase activity
Source: PLoS Genet. 2021 Jun 21;17(6):e1009641. doi: 10.1371/journal.pgen.1009641 (PMC8248709; doi:10.1371/journal.pgen.1009641)
Supplement: S2 Table — (DOCX) [file pgen.1009641.s006.docx]

| **Name/application** | **Sequence 5’-3’** |
| --- | --- |
| sgRNA1-*zPrmt5*-FWD | TAGGGGTGGAACAGCGGCATACAC |
| sgRNA1-*zPrmt5*-REV | AAACGTGTATGCCGCTGTTCCACC |
| genotyping-*zPrmt5*-FWD | CAAGACCTGTCCTGTTTGATGA |
| genotyping-*zPrmt5*-REV | GTGACTTTGCAGGGTCCAGT |
| XhoI-promo*cdh5*-FWD | CCGCTCGAGCCAGGGGCATTTATCTTGG |
| EcorI-promo*cdh5*-REV | CGGAATTCAACGATCGCATACCAGAGT |
| BsaI-distenh*cdh5*-FWD | GTAACGGGTCTCCATGGGACAACAGTCAAAATGTAGCA |
| BsaI-distenh*cdh5*-REV | GTAACGGGTCTCCCTTACACTCGCATAACAATTTCCA |
| BamHI-*gal4VP16*-FWD | CGGGATCCGCCACCATGAAGCTACTGTCTTCTATC |
| SpeI-*gal4VP16*-REV | GGACTAGTCTACATATCCAGAGCGCCG |
| *cdh5*-cloning-FWD | GTCAGTCGGGTCGACGGG |
| *cdh5*-cloning-REV | GTCCTGAACCGAGGTCCCC |
| *fli1a*-cloning-FWD | TCCTCAGCCAGATCCTTATC |
| *fli1a*-cloning-REV | ACCAGCAGAAGGTCAACTTC |
| *runx1*-qPCR-FWD | ACACTGGCGCTGCAACAAG |
| *runx1*-qPCR-REV | CATCATTTCCCGCCATCACT |
| *cmyb*-qPCR-FWD | GAACGGCTACGGTGGCTGGAA |
| *cmyb*-qPCR-REV | CAGAGTCCAGCGAAGGACTGT |
| *rag1*-qPCR-FWD | TGGCATTGAGAGCTGGGAA |
| *rag1*-qPCR-REV | GCCAAACACACTGCAGGATG |
| *rag2*-qPCR-FWD | ACCAAGTACGACTGTGGCTG |
| *rag2*-qPCR-REV | ATGGAGTGCACCTACGTTGG |
| *gata1*-qPCR-FWD | TACTAGTGTGGCAGTTGGCG |
| *gata1*-qPCR-REV | ATGAACGGCCAGAACAGACC |
| *pu.1/sp1*-qPCR-FWD | GCTGGACGTTGTGAGGGTAA |
| *pu.1/sp1*-qPCR-REV | ATTTCATGGACCCAGGCCTG |
| *EF1α*-qPCR-FWD | GATGCACCACGAGTCTCTGA |
| *EF1α* -qPCR-REV | TGATGACCTGAGCGTTGAAG |
| *agtr2*-qPCR-FWD | GTCATGTGCAAGCTGTGTGG |
| *agtr2*-qPCR-REV | AACACATGAACCAACCGGCC |
| *esama*-qPCR-FWD | AGACACCGAGGAGGATCTGG |
| *esama*-qPCR-REV | GCTGGGTTGGTGTTGTATCC |
| *fli1b*-qPCR-FWD | TTCCATCAGCAGTCGTCTTG |
| *fli1b*-qPCR-REV | TAGTTCCCTCCCAGGTGATG |
| *etv2*-qPCR-FWD | TGCCTTTGGAGGAAGAAAGA |
| *etv2*-qPCR-REV | CTGTTGTTGGCAATCTGCTG |
| *cdh5*-qPCR-FWD | CGAGATTGCTGATGGAGGAACGCC |
| *cdh5*-qPCR-REV | TGGCGAGGAGGGCACTGACA |
| *fli1a*-qPCR-FWD | CCAAACATGACGACCAATGAGA |
| *fli1a*-qPCR-REV | GTGATCCGGAGACCACAGAGA |
| *chr7*-neg control-ChIP-qPCR-FWD | ACATCAAAAGTTGACAGAGCAGA |
| *chr7*-neg control-ChIP-qPCR-REV | ATCGCTGGAGACGGTAACAA |
| *cdh5-P*- ChIP-qPCR-FWD | ATCACTTTTGCGTTCGGCTC |
| *cdh5-P*- ChIP-qPCR-REV | GCATTACAGCGCTCAACGAG |
| *cdh5-E*- ChIP-qPCR-FWD | CCCTATTCCCTCCATTAGCCAC |
| *cdh5-E*- ChIP-qPCR-REV | GGCGTGTAATTCCCTACAGGA |
| *fli1b-P*- ChIP-qPCR-FWD | ACTGTTGCCATCCGGTTATG |
| *fli1b-P*- ChIP-qPCR-REV | GAAACGCATCGACTTGTGTG |
| *fli1b-E4*- ChIP-qPCR-FWD | CCCTCAACCTCAAAGCACTC |
| *fli1b-E4*- ChIP-qPCR-REV | TGGGGCATGAGATAGGAAAC |
| *fli1b-E5*- ChIP-qPCR-FWD | TGGCAAAGAGAAGCAGTCAG |
| *fli1b-E5*- ChIP-qPCR-REV | GAAGCAAAGCGGTAAGAAGG |
| *esama-P*-ChIP-qPCR-FWD | CCAGTCATTCCGCTCAGACT |
| *esama-P*-ChIP-qPCR-REV | GCAGACTGACGCTACATCCA |
| *esama-E1*-ChIP-qPCR-FWD | ACTTTTTGGGGTCATTGCAG |
| *esama-E1*-ChIP-qPCR-REV | AATGTCAACAGGCCAGGAAG |
| *esama-E2*-ChIP-qPCR-FWD | TCACAGTCGCTGTTTGGAAG |
| *esama-E2*-ChIP-qPCR-REV | GTGAAGCCCAAGGGACAATA |
| *esama-E3*-ChIP-qPCR-FWD | TCCTCTCGGCTTCTGTTTGT |
| *esama-E3*-ChIP-qPCR-REV | GTGTCTGAAAGGCCAAAGGA |
| *esama-E4*-ChIP-qPCR-FWD | GTTTGACGCGTTCACATGAC |
| *esama-E4*-ChIP-qPCR-REV | GGATGCAGGATCACACACAC |

**S2 Table: Oligonucleotides used in this study.**
