# Supplementary material for: Prmt5 promotes vascular morphogenesis independently of its methyltransferase activity
Source: PLoS Genet. 2021 Jun 21;17(6):e1009641. doi: 10.1371/journal.pgen.1009641 (PMC8248709; doi:10.1371/journal.pgen.1009641)
Supplement: Attachment — Submitted filename: response_reviewers_Plos Genetics_final.pdf [file pgen.1009641.s008.pdf]

First of all, we would like to thank all three reviewers for their useful, constructive and complementary suggestions. We do agree with the reviewers that in order to strengthen our message, we need to consolidate the following three points: (1) PRMT5 catalytic activity is dispensable in blood vessel formation, (2) PRMT5 regulates ETV2 target gene expression *via* a chromatin looping mechanism and (3) PRMT5 is involved in HSC maintenance.

I - PRMT5 activity

As suggested by the 3 reviewers, confirming the status of the methyltransferase activity of Prmt5 in the different contexts of manipulation of Prmt5 (mutant, morphant and rescue experiment) is important to validate our results. Moreover, using a different approach to inhibit Prmt5 activity will support our conclusions concerning the requirement (or not) of its enzymatic activity.

Reviewer 1:

"Demonstration of loss of PRMT5 activity is essential to support the model. Western blot or IF of methylarginine should definitely be performed in the KO, MO, and rescue experiments."

"A western blot or IF of the PRMT5 obligate cofactor MEP50/WDR77 would be a helpful control for knockdown of PRMT5. All evidence in vertebrates points to MEP50 being co-regulated and/or dependent on PRMT5 protein presence"

Reviewer 3:

"3. The major conclusion of this manuscript was that Prmt5 promotes vascular morphogenesis independent of its methyltransferase activity. This was found based on results showed in Figure 4. The main role of PRMT5 is symmetric dimethylation of arginine residues (SDMA), where in most studies, the protein levels of SDMA is usually assessed via western blot. In Figure 4C and D, although a difference is observed between WT and mut Prmt5 mRNA-injected embryos, it would be crucial to look at the SDMA levels to fully state that the methyltransferase activity of Prmt5 is non-essential."

In order to confirm the loss and the rescue of Prmt5 activity, we will perform a panel of WB to analyze the expression of Prmt5, Prmt5 obligate cofactor MEP50/Wdr77, and Prmt5 substrates (using an antibody recognizing symmetric dimethylation of arginine (SDMA)) in wild type and *prmt5* morphant embryos as well as in rescued conditions with *hprmt5WT*- or *hprmt5MUT*. All antibodies are commercially available and were validated in our hands in zebrafish extracts. These WBs are currently being done and will be added to the revised manuscript as supplementary figures.

Of note, mining single cell data (as in Figure S3 in the original manuscript) for *wdr77* expression, we found that *wdr77* is expressed in endothelial cells with similar expression dynamics as *prmt5* (see Figure 1 below). *Wdr77* expression dynamics will be included in the revised manuscript.

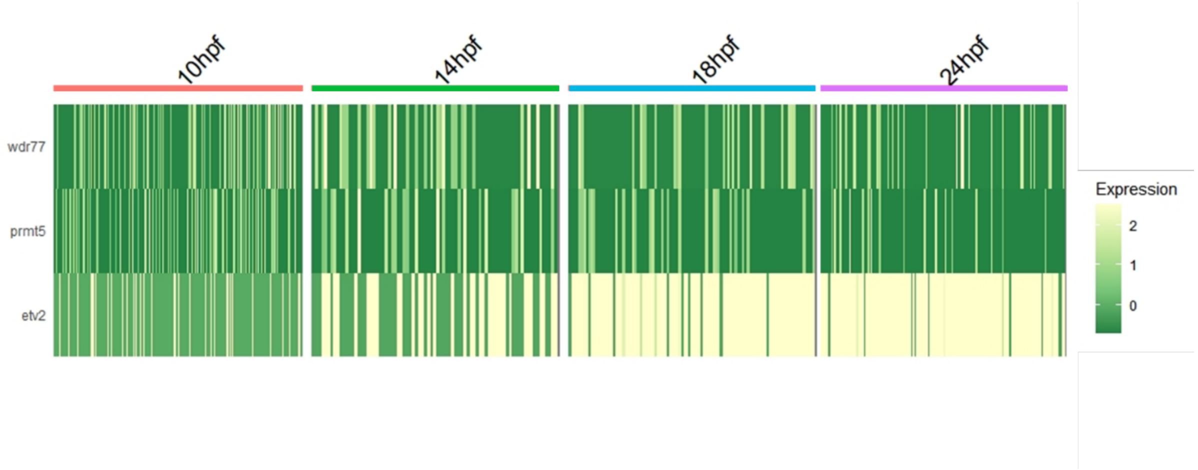

Figure 1 (related to Figure S3 in the original manuscript): Expression heatmap of *wdr77*, *prmt5 and etv2* in endothelial cells at 10hpf, 14hpf, 18hpf and 24hpf. The expression level is colored-coded from absence of expression (green) to highest level of expression (white).

Reviewer 1:

"Use of one of the well documented and potent PRMT5 inhibitors (commercially available at modest cost) would be very helpful to test the model; they could be injected into the embryos"

We agree with reviewer 1 and we have treated zebrafish embryos with EPZ015666, a Prmt5 specific inhibitor, to analyze blood vessel morphogenesis as well as HSC formation with cognate transgenic lines. In line with our model, our **recent results indicate that indeed, inhibition of Prmt5 activity with EPZ015666 impacts lymphoid lineage formation but not blood vessel morphogenesis** (Figure 2).

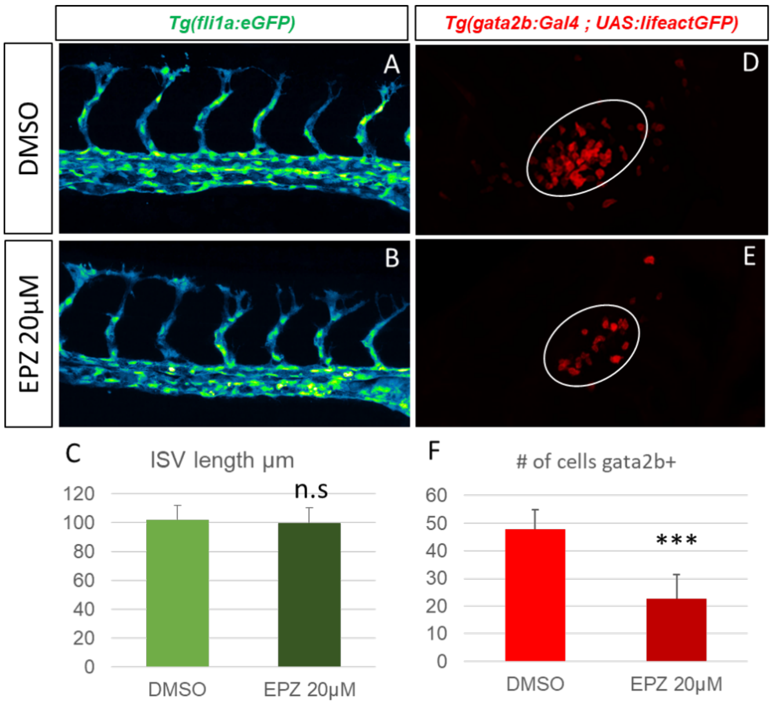

Figure 2- A-B- Confocal projections of transgenic Tg(fli1a:GFP)y1 embryos treated with either DMSO (A) or EPZ 20µM (B) from 9hpf to 28hpf. C- Average Intersegmental vessels (ISV) length in µm, in DMSO and EPZ 20µM treated embryos. D-E- Confocal projections of transgenic Tg(gata2b:Gal4;UAS: lifeactGFP) thymus rudiment from embryos either treated with DMSO (A) or EPZ 20µM (B) from 30hpf to 3dpf. F- Average number of gata2b^{+} cells in the thymus rudiment of DMSO or EPZ 20µM treated embryos. C, F: t-tests were performed (n.s.: not significant; ***: p<0.001)

We also **confirmed the effect of EPZ015666 on Prmt5 activity** by WB using antibodies recognizing symmetric dimethylation of arginine residues (SDMA). All these data will be added to the revised manuscript.

Reviewer 3:

"4. To study if the methyltransferase activity of Prmt5 was required for vascular morphogenesis, the authors utilised human prmt5 WT and mut mRNA. These mRNAs were injected into zebrafish embryos and downstream assessment was performed. However, what is the reason for using human prmt5 mRNA and not zebrafish prmt5 mRNA itself?
Would having cross-species interaction be a concern in the analysis of the results? Perhaps this needs to be clarified."

Reviewer 2:

"Why the authors chose human Prmt5 and human Prmt5 mutant to perform rescue experiment, but not zebrafish prmt5? What are their thoughts?"

In a former study (Batut et al., 2011), we injected human *PRMT5* mRNA in *prmt*5 morphants to avoid the knock-down of the injected *zprmt5* mRNA by *zprmt5* specific morpholino. In these experiments, we found that *hPRMT5* mRNA could fully rescue the *prmt5* Mo phenotype, indicating that PRMT5 function is conserved between human and fish. Thereby, we used the same strategy here.

In addition, by using human PRMT5, we could take advantage of a previously published mutant in the catalytic domain that was shown to abrogate PRMT5 enzymatic activity *in vitro* (Pal et al., 2007). Accordingly, we used it to assess the requirement of PRMT5 activity in our context. To our knowledge, no other catalytic-dead mutant for PRMT5 has been described and validated so far, even in the light of PRMT5 structural data. The extra sets of experiments described above should alleviate any concern on the ability of human mRNA to rescue PRMT5 activity in zebrafish and allow us to establish more firmly that PRMT5 enzymatic activity is dispensable for blood vessel formation.

II- <u>PRMT5, ETV2 and Chromatin looping</u>

II-1: ETV2 and ETV2 target genes regulation

Reviewer 2:

"4. They did not provide any biochemical evidences to show that prmt5 regulates etv2 as post-translation level."

Reviewer 3:

"5. In Figure 3C, it was stated that although ETV2 expression was not altered, its target genes were downregulated in prmt5 mutant. If indeed Prmt5 was modulating ETV2 at the post-translational level, could the authors perform protein analyses such an SDS-PAGE gel and probe for the ETV2 target proteins? They could also analyse the intracellular ETV2 target proteins via FACS if harvesting cells for western blot is a major problem. These experiments will help clarify the speculation of a post-translational modulation by Prmt5. Otherwise, further explanation is required for ETV2's unaltered expression. »

That Etv2 activity could be modulated at post-translational level by PRMT5 (or by PRMT5 coregulators) was just a hypothesis that was mentioned in the text and was not meant to be an important point of the manuscript. We did not explore further this hypothesize as we gained evidence that the methyltransferase activity of Prmt5 was dispensable for blood vessel formation. Thus, the most likely explanation is that PRMT5 does not regulate *etv2* expression but rather is required to regulates Etv2 target gene expression. Nevertheless, it is of interest to determine if the loss of Prmt5 could affect the location of Etv2 in endothelial cells and validate by WB that Etv2 expression is not modified by Prmt5. Therefore, we explored those possibilities by performing WB and IF against ETV2 in control and *prmt5* loss of function contexts. Unfortunately, our recent data indicate that ETV2 antibody from Kerafast (validated for WB application only when ETV2 is *overexpressed*) could not detect *endogenous* ETV2 in our cell extracts by WB. We are currently performing IF experiments with ETV2 antibody. If conclusive, these IF will be added to the revised version of the manuscript and the results will be discussed accordingly.

II-2: Chromatin looping

Data mining as well as our analyses of transgene expression with different chromatin architecture in different conditions led us to propose that PRMT5 promotes chromatin looping in endothelial cells. Further genomic experiments, as recommended by reviewer 1, could undoubtedly strengthen our model.

Reviewer 1:

"The H4R3me2s ChIP-Seq studies in the literature have not been widely confirmed, and there are reasons to suspect that those antibodies recognize non-histone proteins. Many recent mass spec reports show that many chromatin associated proteins have methylarginine. Therefore, the elaborate analysis in Table 1 must be viewed with substantial skepticism. Indeed, independent tests of this hypothesis with ETV2 and histone methylarginine chip-qpcr are probably warranted. If this is too technically challenging in fish embryos, then the authors should consider alternative approaches to directly support the chromatin based model."

Thanks for the very useful information about the concerns related to the use of H4R3me2s antibodies in ChIP-seq studies. Indeed, we were not aware of the suspicion around their specificity.

Performing ChIP-qPCR experiments with Etv2 and methylarginine antibodies as suggested by the reviewer cannot be an option as (1) we would need to use methylarginine antibodies that could again raise specificity issues and (2) antibodies against zebrafish Etv2 have not been validated in ChIP-qPCR experiments.

Rather, since we propose that Prmt5 might help to contribute to chromatin looping at specific Etv2 target genes in endothelial cells, we will assess by ChIP-qPCR the binding of Prmt5 on promoters and putative enhancers in genes of interest, such as *esama* or *cdh5*. We will also perform ChIP-qPCR against two factors that known to facilitate enhancer-promoter interactions in cooperation with Prmt5: Brg1 -the ATPase of the mammalian SWI/SNF chromatin remodeling complex- and/or Med1 -a subunit of Mediator complex-(LeBlanc et al, 2016). The co-localization of Prmt5 with Brg1 and/or MED1 on promoters and enhancers would support the formation of DNA loops at these loci.

III- HSC maintenance

Reviewer 3 also suggests to provide more evidence that Prmt5 is required for HSC maintenance in zebrafish and to further investigate the impact of *prmt5* loss of function on hematopoietic lineages.

Reviewer 3

"1.For the first part of the study, the authors claimed that Prmt5 is required for HSC maintenance. However, this claim was made only on the basis that Prmt5 mutant embryos had an increased number of gata2b+ HSCs. This may not be sufficient. Similar to other studies that have made conclusions regarding HSC maintenance, perhaps the authors could assess the cell cycle distribution of HSCs to evaluate how the HSC compartment is affected. Furthermore, to strengthen the notion that Prmt5 is important for HSC maintenance in zebrafish, it might be useful to pharmacologically (small-molecule inhibitor) or genetically (siRNA/shRNA) inhibit Prmt5 in zebrafish cell lines (ZF4, ZEM2S). These experiments would aid in conclusively stating that Prmt5 is indeed essential for HSC maintenance in zebrafish."

In order to further characterize the role of Prmt5 during blood cell development and in particular in HSC proliferation and maintenance, we will assess the percentage of HSCs and HSPCs in mitosis by IF against Phospho Histone H3 and by imaging the dorsal aorta and the CHT in control and *prmt5* morphant embryos, respectively. It was shown in mouse that *Prmt5* loss of function prevents the progression of HSPCs through the cell cycle but promotes HSCs proliferation (Liu et al., 2015). As shown below (Figure 3), our recent analysis of the number of gata2b+ HSPCs in CHT of control embryos and *prmt5* morphants at 3 days suggests that **Prmt5 is required to maintain the appropriate number of HSPCs in zebrafish**. Additional cell cycle information will provide a deeper insight into the role of Prmt5 on those cells and if it is conserved through evolution. These data will be integrated in the revised manuscript.

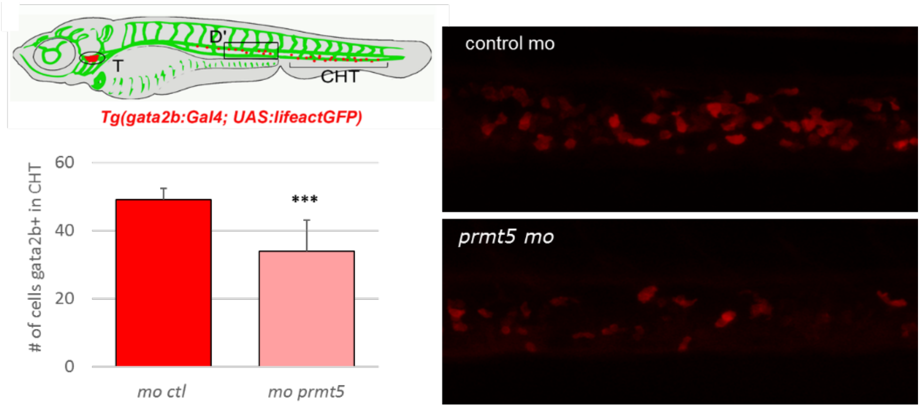

Figure 3: Impact of PRMT5 loss on gata2b^{+} CHT development.

Of note, the zebrafish cell lines ZF4, ZEM2S are fibroblast cells and, to our knowledge, there is no available protocol describing their use for assessing HSCs or HPSCs maintenance or expansion.

"2. Similarly, the statement that Prmt5 is required for lymphoid progenitor expansion is also loosely made based on their finding that lymphoid progenitors were reduced in Prmt5 mutants. The expression of B cells should also be assessed as well as T cells."

We agree with reviewer 3 that having more information on the fate of the different hematopoietic progenitors would improve our study on this aspect. To this end, we performed RT-qPCR for specific key markers of the different cell lineages such as common lymphoid progenitor markers (*rag1, ikaros)*, T cell markers (*rag2, lck*), erythrocyte markers (*gata1, hbaa),* or myeloid markers *(pu1, mpx)* (Stachura et al., 2009; Berrun et al., 2018; Chi et al., 2018; Rasighaemi et al., 2015). With these experiments, we found a reduction of expression of lymphoid progenitor markers in *prmt5* loss of function context as compared to control embryos. Also, we observed an increase of expression of erythrocyte markers but not of myeloid markers, suggesting that the loss of *prmt5* promotes the production of erythrocyte but does not affect myelopoiesis. These data will be integrated in the revised manuscript.

IV – Other points

Reviewer 1:

"On line 92, the authors state that PRMT5 is known to mainly repress transcription. This is not true: many reports show that PRMT5 activates transcription"

"I suggest that relative expression in qRTPCR experiments be transformed as a log2FC. Otherwise the data is not normally distributed and the ANOVA (a parametric test) is inappropriate. E.g. Fig 1H, 3C, 4G"

We agree with reviewer 1 and will change the text and the figures, accordingly.

"As PRMT5 is universally expressed in every vertebrate cell tested, and appears to be universally expressed in fish embryos (e.g. Fig 2A-C), it is unclear the value of this experiment. What are the authors learning by showing that PRMT5 is everywhere? Again, probing PRMT5 cofactors may be of value, or, more significantly, the methylarginine product of PRMT5 (e.g. general Rme2s antibodies or histone methylarginine specific antibodies)"

As mentioned above, we will inspect Prmt5 target proteins and cofactors in the different conditions to further validate our approaches. We are also considering performing IF against the mentioned proteins to gain insight on where and when Prmt5 activity is required. As for the Fig 2A-C, we wanted to inspect when and precisely in which endothelial cells Prmt5 is expressed even though Prmt5 is thought to be ubiquitously expressed. By doing so, we ruled out that there was a preferential expression of Prmt5 in tip or stalk cells in ISVs for instance, which could have led us to different interpretations of our data. Also those IF experiments confirmed the scRNA-seq data analysis since we noticed a higher endothelial expression of Prmt5 in early zebrafish embryos as compared to older ones. We will modify the text accordingly.

Reviewer 2:

"1.As described in this manuscript, disruption of prmt5 in zebrafish causes obvious defects in hematopoiesis and blood vessel formation, what is the general phenotypes displayed in adult prmt5-dificient zebrafish? Any general phenotypes related to defects in hematopoiesis and blood vessel formation?"

In a recent study demonstrating the role of Prmt5 during germ cell development in zebrafish (Zhu et al., 2019), it was shown that *prmt5* mutants exhibited a lower survival rate 15 dpf (18% instead of 25% for wild type siblings) but without any further investigation. It would be of course, an interesting point to address, but we believe the study in adults is out of the scope of the present manuscript.

"2.Why the phenotypes displayed in prmt5-null zebrafish (Fig.4) is not as dramatic as those displayed in prmt5 morphants (Fig.5)? Based on Fig.4, it is very difficult to judge the rescue effect."

First, we will change the panel of the wild type condition in Figure 4 with a more representative picture (Panel A, figure below) in the revised manuscript.

figure4

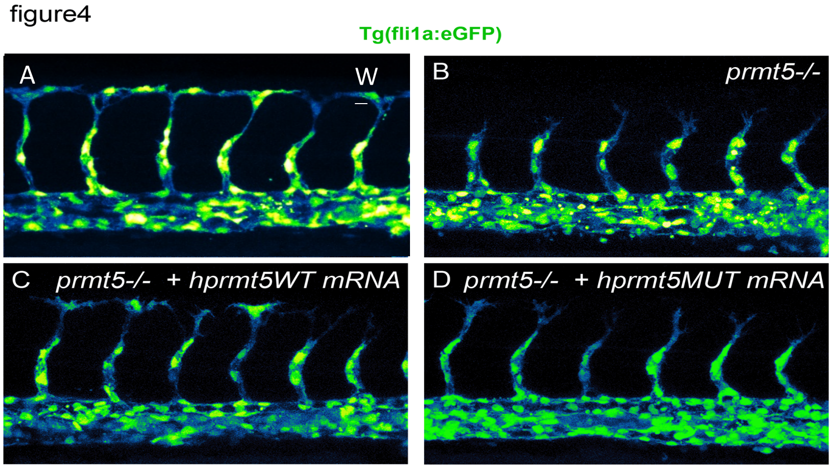

"5.What is the reason why the regulation of most ETS genes is independent of prmt5 enzymatic activity, but the regulation of fli1a is dependent of prmt5 enzymatic activity?"

As evoked in our discussion, we did not observe any distant enhancer in *fli1a* genomic landscape suggesting that the proposed chromatin looping function of Prmt5 (independent of its enzymatic activity) will not be required for this gene. In that case (*i.e.* without looping), Prmt5 could modulate gene expression through its "classical" enzymatic-dependent activity. It is interesting to note that unlike other identified Prmt5 target genes, *fli1a* is expressed concomitantly with *etv2* and *prmt5* (data derived from single cell RNA-seq dataset in Figure S3), which could indicate that the regulation of *fli1a* expression might rely on yet unknown different mechanism(s).

Reviewer 3

"2. In figure 2, perhaps figures 2J and K could be swapped so that the order of the results matches the flow of the manuscript text."

"3. Could the resolution of images used in Figure 2 (A, B, C, E, F, G) be improved for better clarity?"

We agree with reviewer 3 and will change the text and the figures (in higher resolution) accordingly.
