# Supplementary material for: Prmt5 promotes vascular morphogenesis independently of its methyltransferase activity
Source: PLoS Genet. 2021 Jun 21;17(6):e1009641. doi: 10.1371/journal.pgen.1009641 (PMC8248709; doi:10.1371/journal.pgen.1009641)
Supplement: Attachment — Submitted filename: Reviewer3_10may_v2.pdf [file pgen.1009641.s009.pdf]

Reviewer 3

We would like to thank reviewer 3 for his/her useful comments. We have now added 2 full figures (Figure 2 and Figure S1) to clarify the points raised and to strengthen the role of Prmt5 in hematopoiesis. In addition, extensive validation of the rescue experiments combined with the use of a drug inhibitor further support our conclusions that in contrast with blood cell formation, vascular morphogenesis is independent on Prmt5 methyltransferase activity.

The main finding of this manuscript is that Prmt5 is able to promote vascular morphogenesis via the transcriptional regulation of ETS and adhesion proteins. Intriguingly, the authors claimed that the methyltransferase activity of Prmt5 was non-essential for the regulation of gene expression and hence proposed that Prmt5 might be acting as a scaffold protein to facilitate chromatin looping; in endothelial cells. Using various zebrafish transgenic lines such as Tg(gata2b: Gal4;UAS:lifeactGFP) and Tg(fli1a:eGFP), the authors were able to visually study the impact of Prmt5 on HSC maintenance and vascular morphogenesis. Previously published big datasets like single cell RNA-sequencing and ATAC-seq were mined for novel insights pertaining to this study and allowed the authors to make conclusive statements. Despite these, further clarifications are required for some parts of the study in terms of mechanisms relating to Prmt5 and the transcription factor ETV2. Also, the first part involving HSC maintenance needs more analyses to support the finding. The specific comments are as listed below;

Major Comments:

1.For the first part of the study, the authors claimed that Prmt5 is required for HSC maintenance. However, this claim was made only on the basis that Prmt5 mutant embryos had an increased number of gata2b+ HSCs. This may not be sufficient. Similar to other studies that have made conclusions regarding HSC maintenance, perhaps the authors could assess the cell cycle distribution of HSCs to evaluate how the HSC compartment is affected. Furthermore, to strengthen the notion that Prmt5 is important for HSC maintenance in zebrafish, it might be useful to pharmacologically (small-molecule inhibitor) or genetically (siRNA/shRNA) inhibit Prmt5 in zebrafish cell lines (ZF4, ZEM2S). These experiments would aid in conclusively stating that Prmt5 is indeed essential for HSC maintenance in zebrafish.

Thanks for the suggestions. However, we would like to point out that the first part of the study on hematopoiesis was mainly an « entry point » to set up conditions and determine whether Prmt5 was regulating hematopoiesis in zebrafish as it has been shown in mouse (Liu et al, JCI, 2015), before analyzing more thoroughly Prmt5 function in blood vessel formation.

Nevertheless, we performed experiments to further characterize the role of Prmt5 during blood cell development and in particular its impact on HSC proliferation (by analyzing phospho H3 staining in HSC) and maintenance (by analyzing gata2b+ progenitor number in the caudal hematopoietic tissue in addition to the thymus). We also used a pharmacological inhibitor to confirm our data (please see below our response to the next point). The corresponding new data have been added pages 6-7 and in figure S1 in the revised manuscript:

"We first confirmed that prmt5 loss-of-function by prmt5-specific morpholino injection resulted in a similar increase of HSCs as compared to control morphants (Fig S1A). In order to further characterize the role of Prmt5 in HSC proliferation, we assessed the percentage of HSCs (gata2b+ cells) in mitosis by co-immunostaining for Phospho Histone H3 (P-H3). We found that, as in mouse (Liu et al, JCI, 2015), the increase of HSC number was associated with enhanced proliferation in prmt5 morphants as compared to control embryos (10.15% versus

1.92% of gata2b+, P-H3+ cells) (S1B, C Fig). Altogether, these results suggest that Prmt5 regulates the number of emerging HSCs from the dorsal aorta. We next investigated whether blood cell formation was impaired in prmt5 zebrafish mutant as described in mouse (Liu et al, JCI, 2015). HSPC give rise to different blood cell progenitors, such as lymphoid progenitors which colonize the thymus leading to T lymphopoiesis, but also erythroid or myeloid progenitors that will home in the CHT (Fig 1D) (Ma et al, 2013). We found that at 5 days, the number of gata2b+ HSPCs in the thymus was significantly reduced in prmt5 mutant as compared to wild-type (Fig 1I-K). Similarly, imaging the caudal vascular plexus of prmt5 morphant and control embryos at 3 dpf showed a reduced number of gata2b+ HSPCs in the CHT of prmt5 morphants. Thus, as in mouse (Liu et al, JCI, 2015), Prmt5 seems required for HSPC maintenance in zebrafish (S1D-G Fig).
Hence, altogether, our data indicate that as in mouse (Liu et al, JCI, 2015), Prmt5 loss-of-function prevents the progression of HSPCs through the cell cycle but promotes HSCs proliferation and supports an important and conserved role of Prmt5 in hematopoiesis. »

Furthermore, to strengthen the notion that Prmt5 is important for HSC maintenance in zebrafish, it might be useful to pharmacologically (small-molecule inhibitor) or genetically (siRNA/shRNA) inhibit Prmt5 in zebrafish cell lines (ZF4, ZEM2S). These experiments would aid in conclusively stating that Prmt5 is indeed essential for HSC maintenance in zebrafish.

Please, note that the zebrafish cell lines ZF4, ZEM2S are isolated from early staged embryos and present a fibroblastic morphology. To our knowledge, there is no evidence and no published protocols describing the culture of HSCs derived from those cells.

However, to fully support that Prmt5 is important for HSC maintenance in a methyltransferase activity-dependent way, we performed a panel of analyses combining morpholino-based loss-of-function with rescue experiments using either a wild-type or a catalytic dead point mutant of *prmt5* mRNA, as well as treatment of embryos with a specific inhibitor of Prmt5 activity (EPZ015666). These experiments confirmed the specificity of the loss-of-function phenotypes we described and the importance of Prmt5 enzymatic activity for hematopoiesis in zebrafish. The corresponding data are described pages 8-9 (see below) and displayed on Figure 2.

"However, to determine the impact of Prmt5 methyltransferase on the fate of gata2b+ cells in the thymus, we combined morpholino-based loss-of-function with rescue experiments using either a wild-type or a catalytic dead point mutant of human prmt5 mRNA, all of which have been previously validated (16,23,31). To first validate the effect of prmt5 morpholino and the respective rescues, immunostainings were performed on 5 hpf embryos to detect either Prmt5, its obligate co-factor Mep50/Wdr77 or symmetric dimethylarginines (the generic product of arginine methylation by Prmt5 and some other Prmts) on control embryos, prmt5 morphants and prmt5 morphants injected with wild-type or with catalytic point mutant prmt5 mRNA (Fig 2A-L). As expected, Prmt5 expression was strongly decreased in prmt5 morphant and was rescued after injection of prmt5 wild type or point mutated mRNAs (Fig 2A-D). Interestingly, Mep50/Wdr77 expression followed the same trend as Prmt5, supporting the finding that they regulate each other (32)(Fig 2E-H). Symmetric dimethylation levels slightly decreased in prmt5 morphants as compared to control embryos and strongly increased upon injection of wild type prmt5 mRNA but not of prmt5 mutant mRNA (Fig 2I-L). This confirms that prmt5 wt mRNA rescues efficiently Prmt5 enzymatic activity while prmt5 mRNA mutated form is indeed coding a catalytic dead Prmt5 protein. Altogether, these data validate our approach as we observed the loss of Prmt5/Mep50 complex following prmt5 morpholino injection, the efficient rescue of the complex and of its enzymatic activity by prmt5 wt mRNA, and the rescue of the complex but not of its enzymatic activity with prmt5 catalytic mutant mRNA. Moreover, Prmt5 immunostaining in 24 hpf embryos validated the efficiency of prmt5 loss-of-function and rescues at later stage (Fig 2M-P). Hence, we used this approach to determine whether Prmt5 enzymatic activity was required for the formation of gata2b+ cells in the thymus at 3 dpf using

the Tg(gata2b:Gal4;UAS:lifeactGFP) line (Fig 2Q-T). We found that gata2b+ cell number was reduced in prmt5 morphants as compared to control embryos and this effect was rescued upon injection of prmt5 wt mRNA but not of prmt5 catalytic mutant mRNA (Fig 2Q-T, X). These data underscore the conserved requirement of Prmt5 in hematopoiesis among species, as human prmt5 mRNA could efficiently rescue Prmt5 function in prmt5 morphants, as previously shown in myogenesis (23). More importantly, they also show that Prmt5 enzymatic activity is required for gata2b+ cell formation in the thymus. To further support these findings, we assessed the impact of the well-established Prmt5 specific inhibitor (EPZ015666) on wild-type zebrafish embryos. EPZ015666 treatment efficiently inhibited Prmt5 activity in vivo as increasing concentrations of this drug led to a dose-dependent decrease of global symmetric dimethylarginines, as seen by western blot (Fig 2W). Interestingly, we found that EPZ015666 treatment also led to a decrease of gata2b+ cell number which was similar to the one observed in prmt5 morphant (Fig 2U, V, X). Hence, our data show that Prmt5 enzymatic activity is required for its function in hematopoiesis".

2.Similarly, the statement that Prmt5 is required for lymphoid progenitor expansion is also loosely made based on their finding that lymphoid progenitors were reduced in Prmt5 mutants. The expression of B cells should also be assessed as well as T cells.

To address this point, we performed RT-qPCR in control and *prmt5* morphants to assess the expression of different blood cell markers. These data are now described page 7 (see below) and displayed in figure S1H-K.
"Analyzing the expression of the common lymphoid progenitor marker rag1 and of the mature T cell marker rag2 showed that both genes were downregulated in prmt5 morphant as compared to control embryos (S1H, I Fig). In contrast, the expression of the erythrocyte marker gata1 was increased and that of the myeloid marker spi1/pu.1 was not affected (S1J, K Fig). These data suggest that prmt5 loss impairs lymphoid progenitors and T cell differentiation but promotes the production of erythrocyte without affecting myelopoiesis. In sum, our data support the conclusion that Prmt5 plays an important and conserved role for hematopoiesis in vertebrates".
Of note, expression of B cells could not be assessed as they differentiate much later (at 3 weeks post-fertilization).

3.The major conclusion of this manuscript was that Prmt5 promotes vascular morphogenesis independent of its methyltransferase activity. This was found based on results showed in Figure 4. The main role of PRMT5 is symmetric dimethylation of arginine residues (SDMA), where in most studies, the protein levels of SDMA is usually assessed via western blot. In Figure 4C and D, although a difference is observed between WT and mut Prmt5 mRNA-injected embryos, it would be crucial to look at the SDMA levels to fully state that the methyltransferase activity of Prmt5 is non-essential.

As mentioned above, we validated our approaches by a panel of IFs to analyze the expression of Prmt5, its obligate cofactor MEP50/Wdr77, and its substrates (using an antibody recognizing symmetric dimethylarginine (SDMA)) in wild type and *prmt5* morphant embryos as well as in rescued conditions with *prmt5WT*- or *prmt5MUT*. The corresponding text and data have been added to the revised manuscript (page 8 and Figure 2A-L).
Concerning the requirement (or not) of PRMT5 enzymatic activity, we have treated zebrafish embryos with the inhibitor EPZ015666 and analyzed blood vessel morphogenesis. In line with our model, we found that inhibition of Prmt5 activity with EPZ015666 did not impact blood vessel development (Figure 4 E, F, G, H in the revised manuscript). Of note, the effective inhibition of PRMT5 activity upon embryo treatment with EPZ015666 was confirmed by WB using an antibody against SDMA (Figure 2W in the revised manuscript).

4.To study if the methyltransferase activity of Prmt5 was required for vascular morphogenesis, the authors utilised human prmt5 WT and mut mRNA. These mRNAs were injected into zebrafish embryos and downstream assessment was performed. However, what is the reason for using human prmt5 mRNA and not zebrafish prmt5 mRNA itself? Would having cross-species interaction be a concern in the analysis of the results? Perhaps this needs to be clarified.

In a former study (Batut et al., 2011), we injected human *PRMT5* mRNA in *prmt*5 morphants to avoid the knock-down of the injected *zprmt5* mRNA by *zprmt5* specific morpholino. In these experiments, we found that *hPRMT5* mRNA could fully rescue the *prmt5* Mo phenotype, indicating that PRMT5 function is conserved between human and fish. Thereby, we used the same strategy here.
In addition, by using human PRMT5, we could take advantage of a previously published mutant in the catalytic domain that was shown to abrogate PRMT5 enzymatic activity *in vitro* (Pal et al., 2007) and *in vivo* in mice (see for example: Liu et al., JCI, 2015). Accordingly, we used this mutant to assess the requirement of PRMT5 activity in our context. To our knowledge, no other catalytic-dead mutant for PRMT5 has been described and validated so far, even in the light of PRMT5 structural data. Our strategy is now clarified in the text of the revised manuscript (page 8). With the extra sets of experiments described above with the EPZ015666 inhibitor, it seems reasonable to conclude that human mRNA can rescue PRMT5 activity in zebrafish and that PRMT5 enzymatic activity is indispensable for blood cell formation but dispensable for blood vessel formation in this model organism.

5.In Figure 3C, it was stated that although ETV2 expression was not altered, its target genes were downregulated in prmt5 mutant. If indeed Prmt5 was modulating ETV2 at the post-translational level, could the authors perform protein analyses such an SDS-PAGE gel and probe for the ETV2 target proteins? They could also analyse the intracellular ETV2 target proteins via FACS if harvesting cells for western blot is a major problem. These experiments will help clarify the speculation of a post-translational modulation by Prmt5. Otherwise, further explanation is required for ETV2's unaltered expression.

That Etv2 activity could be modulated at post-translational level by PRMT5 (or by PRMT5 coregulators) was only a hypothesis and was not meant to be an important point of the manuscript. We did not explore further this hypothesize as we gained evidence that the methyltransferase activity of Prmt5 was dispensable for blood vessel formation. Since PRMT5 does not regulate *etv2* expression, we favored the hypothesis that it is required to regulate Etv2 target gene expression, but indeed we cannot rule out that it might somehow affects Etv2 protein level or localization. In line with the reviewer suggestions, we explored those possibilities by performing WB and IF against Etv2 in control and *prmt5* loss-of-function contexts. Unfortunately, our data indicate that Etv2 antibody from Kerafast (validated for WB application only when Etv2 is *overexpressed*) could not detect *endogenous* Etv2 in zebrafish cell extracts by WB. Besides, despite several trials of IF experiments with Etv2 antibody, we have not been able to detect any specific signal. Thus, to avoid any confusion, we removed the hypothesis concerning ETV2 post-translational modification by PRMT5 from the text and changed it into:
"*As expected, prmt5 expression was reduced in the mutant but etv2 expression was not affected (Fig 4I, J). However, the expression of ETS transcription factors (fli1a, fli1b) and adhesion proteins (cdh5, agtr2 and esama), all putative Etv2 target genes (6,7), was significantly reduced in prmt5 mutant (Fig 4K). Of note, the reduction of fli1a and cdh5 expression as detected in prmt5 mutant by RT-qPCR was confirmed by in situ hybridization in the hemogenic endothelium (S3J-S Fig). That etv2 expression was unaffected by the loss of*

prmt5 whereas its targets were down-regulated suggests that Prmt5 is required to promote Etv2 target gene expression" (page12 in the revised manuscript).

Minor Comments:

1.The choice of appropriate transgenic models for the various parts of the study was well made. Especially in figures 2F and 3B where the defect in ISV sprouting and lumen formation were very clearly depicted.

Thanks for your positive comment.

2.In figure 2, perhaps figures 2J and K could be swapped so that the order of the results matches the flow of the manuscript text.

We have swapped the two panels, as suggested (Figure 3 panels H and I in the revised manuscript).

3.Could the resolution of images used in Figure 2 (A, B, C, E, F, G) be improved for better clarity?

We cannot improve the quality of the figure as the magnification is very high, but we think that the « non-compressed » version will be of better resolution. Please, also note that we moved the panels A-D of the original Figure 2 to the figure S2A-D in the revised manuscript as the immunofluorescence experiments with PRMT5 showed a ubiquitous expression and several new important experiments had to be included in the main figures of the revised version.
